# Pathology and Practice of Medicine

**Published:** 1860-07

**Authors:** Richard J. Dunglison


### PATHOLOGY AND PRACTICE OF MEDICINE.

BY RICHARD J. DUNGLISON, M.D.,

I.—*Diagnosis and Treatment of Special Forms of Paralysis, with illustrative Cases.*

Several interesting theoretical and practical views in regard to paralytic affections having recently appeared in the journals of this country and Europe, it is deemed expedient to arrange them in a series, in order to furnish a convenient mode of comparison of the peculiarities of each.

1. *Diagnosis and Treatment of the Principal Forms of Paralysis of the Lower Extremities.* By C. E. BROWN-SÉQUARD, M.D., etc. Lecture I. Parts I., II., and III. (London Lancet, April 21, 28, and May 5 )

The object of this first lecture of Dr. Brown-Séquard is to establish a clear diagnosis between the different forms of paralysis, and especially of the two most frequent and distinct forms, reflex paraplegia and the paralysis due to myelitis. The great difference in the mode of treatment of these two affections renders a differential diagnosis of the utmost importance. *Reflex paraplegia* is defined as "a paralysis of the lower limbs due to an excitation that has come to the spinal cord from a sensitive nerve; the excitation, after having reached this nervous centre, may be *reflected* on the blood-vessels of this very centre, or on those of the motor nerves or the muscles." Dr. Brown-Séquard advances two propositions: First, that a paralysis of the lower limbs may be caused by an alteration in the periphery or the trunk of the various sensitive nerves; second, that this kind of paralysis differs extremely from the other kinds of paraplegia in many of its symptoms, and in the frequency and rapidity of cure. Many of the characteristics of reflex paralysis are described with a view of showing how distinct it is from cases of paralysis dependent upon an evident organic alteration of the nervous centres, and to confute the arguments of those who deny the existence of such a condition as reflex paraplegia. A long list may be given of cases of paralysis, the production of which cannot be explained otherwise than by a reflex action, such as amaurosis, or paralysis of the optic nerve, due to injury of the frontal nerve, which has been cured rapidly after the section of the nerve between the irritated spot and the nervous centre, so as to abolish reflex action; paralysis of one arm, one hand, the eye, the œsophagus, etc., consequent upon an excitation of a sensitive nerve of animal or organic life; paralysis of the auditory nerve as a consequence of neuralgia, appearing, increasing, diminishing, or disappearing with this outside nervous excitation, etc.

The difference between reflex paraplegia and paraplegia from myelitis is fully illustrated by the lecturer in fifteen of the prominent symptoms of paraplegia from diseases of the urinary organs and paraplegia from myelitis, deduced from cases detailed by Messrs. Stanly, Rayer, and others. The treatment of reflex paraplegia must aim at the cure of the disease which has caused the irritation, as in those cases which are dependent upon diphtheria, or enteritis, or neuralgia, or disease of the knee-joint, or nephritis. Illustrations of each form are detailed by Dr. Brown-Séquard, in which there existed no reason for believing that the affection had its origin primarily in any disease of the spine or its contents. Suppress the external cause of irritation, and the reflex paraplegia will often be cured, while all treatment directed merely to the relief of the paralysis, without considering its reflected character, will fail. Many, however, doubt the necessity of admitting this view of the existence of reflex paraplegia from external irritation; in cases of disease of the kidney, for example, paraplegia may be due to uræmic poisoning; in diphtheria or disease of the lungs, to an alteration in the blood, caused by the disturbance of digestion or respiration; in disease of the uterus by that organ pressing upon the obturator nerve and the sacral plexus, etc. But Dr. Brown-Séquard examines into all these difficulties, individually. Uræmic poisoning does not, for instance, manifest itself only by a paralysis, but excites other very striking symptoms, which were absent entirely in these cases; the alteration of the blood cannot account for diphtheritic paralysis, for it is impossible to suppose that a complete paraplegia, without any diminution of voluntary movement elsewhere, can be due to a cause, which, as it is supposed to be in the blood, is circulating everywhere in the body; nor can pressure of the uterus upon the nerves of the lower limbs be the cause of the paralysis, unless the organ has undergone great enlargement. He thinks it is conclusively proven that the period of appearance, the variations of intensity, the progress and the cure of paraplegia, are in perfect correspondence with the view that the paralysis depends upon an irritation from a sensitive nerve.

The remainder of this lecture is occupied in the discussion of the two interesting topics of reflex contraction of vessels, and the morbid reflex action on nutrition, and in the consideration of the treatment of reflex paraplegia. Being accompanied and most likely produced by an insufficient amount of blood in the spinal cord, it ought not to be treated by those remedies which diminish the quantity of this fluid in the spinal nervous centre. How often, however, is mercury, which acts in this way, employed in that affection! On the other hand, myelitis, which is accompanied by an increase in the quantity of blood in the spinal cord, is often treated by the very remedy (strychnine) which has the most powerful influence in increasing the amount of blood in the spinal cord! How important, therefore, is it to know exactly to which group of affections (as to the quantity of blood in the spinal cord) belongs a paraplegia, before we give a remedy included in either of the two categories above mentioned!

2. *Paralysis Consequent upon the Poison of Diphtheria.* By James B. Reynolds, M.D., etc. (N. Y. Journal of Medicine, May, 1860.)

*Diphtheritic paralysis* has not been recognized or well described as a distinct disease consequent upon the poison of diphtheria until of late years; and many cases which were mentioned during the last century as curious deviations from special forms of sore-throat, may be considered as those which would now form illustrations of diphtheritic paralysis. It always begins in the soft palate; and, if the eyes are affected, amaurosis occurs, followed perhaps by deafness; then the lower limbs, the upper extremities, the muscles of the alimentary canal and bladder, etc., become affected; paralysis being sometimes confined to loss of motion, or the nerves of sensation merely. The invasion of these different parts comes on slowly and gradually, and in most of the cases every trace of the primary affection has disappeared. The various forms of paralysis, and especially the affections of the throat and eyes, are fully described by Dr. Reynolds. In one case total blindness existed for three months, but the patient recovered without any indistinctness of vision; yet in a large number of these amaurotic cases, no lesion could be detected by the ophthalmoscope. Disturbance of the organ of hearing is rare, paralysis being often, indeed, extended no further than to impairment of the functions of the palate and organs of vision. There may be merely muscular feebleness of the extremities, or total paralysis may exist. When the muscles of the face are involved, as sometimes, though rarely, happens, the expression of the countenance becomes altered, often assuming a hideous appearance from the unequal want of power in the two sides of the face. In these severe cases, obstinate constipation occurs from the paralysis of the muscular coat of the intestines; in one case no action of the bowels could be effected within twenty-three days. The muscular fibres of the heart are very rarely attacked. A very slight primary affection may be followed by most serious sequelæ.

The symptoms thus detailed are those involving the motor system, but some of the very earliest phenomena are connected with more or less modification of the sensory system. Anæsthesia of one leg sometimes occurs, with hyperæsthesia of the other; and it occasionally happens that where the paralysis of motion is nearly or quite the same in both legs, sensation may be altered in but one. The mind is but rarely affected. Cases of diphtheritic paralysis are often confounded with the results of hysteria or general progressive paralysis, with idiocy, etc. The prognosis is favorable; only nine of the seventy-seven cases referred to by Dr. Reynolds died. Death resulted from convulsions, starvation, syncope, and from the passage of masses of meat into the trachea, etc. The main cause assigned by Bretonneau for the production of the paralysis is the extension of the false membrane up through the nasal fossæ; but this view is regarded as untenable. M. Trousseau has abandoned his former opinion, that loss of power was dependent on inflammation of the coats of the nerves, and on infiltration producing pressure on their motor muscles, and now believes it to be the direct consequence of the diphtheritic poison acting generally on the system and strangely modifying the blood; a view which has been already touched upon in the abstract of Dr. Brown-Séquard's lecture. The treatment is such as would readily suggest itself to the therapeutist, embracing as it does the use of tonics, friction, and special nervous stimulants.

3. *Paralysis of Motor Power in one Leg, and of Sensibility in the other.* By SAMUEL CHEW, M.D., etc. (Maryland and Virginia Medical Journal, April, 1860.)

A case admitted into the Baltimore Infirmary presented impairment of sensibility alone in one leg, and of the power of motion alone in the other. Such cases are probably rare. Dr. Chew explains them according to the results of the experiments and researches of Dr. Brown-Séquard, and considers that they probably arise from injury inflicted upon some portion of one lateral half of the spinal cord, as by the pressure of a tumor, by congestion or effusion of blood, or by the changes caused by inflammation. Injury of either lateral half of the cord occasions, in all cases, complete paralysis of *motor power* in the *corresponding* side of the body below the point of injury; but Dr. Brown-Séquard has discovered, also, that such an injury occasions equally complete paralysis of *sensibility* in the *opposite* side. Such a case as that detailed by Dr. Chew occurs but rarely, because almost all the accidents that can befall the cord are, from their nature, far more apt to affect both its lateral halves than either of them alone.

4. *Paralysis of the Right Side, and very rapid Recovery.* By C. J. B. ALDIS, M.D. (Medical Times and Gazette, March 31, 1860.)

Dr. Aldis was called, in February last, to see a lady of sixty-nine, in consequence of her having become speechless. She was conscious, but had flapping of the buccinator muscle and hemiplegia of the right side; the tongue, when protruded, inclining to the paralyzed side. The right arm and leg dropped like a stone, after being raised. While a mustard-poultice was being prepared, she was noticed to point to the toes of the right foot, and it was observed that strength was gradually returning to the leg and other parts of the affected side, so that in twenty minutes the paralysis had entirely ceased. Altogether not more than thirty-five minutes had elapsed between the commencement of the attack and its spontaneous cure. She had been subject to intermittent pulse for several years, but there was no organic disease of the heart. The prognosis in a case in which the symptoms indicated an affection of the fifth and ninth nerves, with loss of power over the whole of the right side, could not, in ninety-nine cases out of a hundred, be favorable; and some lesion of the brain, either by softening or effusion, connected with diseased arteries, might naturally have been suspected.

It is probable, however, as Dr. Aldis thinks, that venous congestion of the brain, caused in a great measure by the loaded state of the bowels, with a weak heart, excited the symptoms, which, fortunately, were only transient. [Two similar cases are described by Mr. Ewens, in *Med. Times and Gaz.*, April 14, 1860; one of which terminated fatally during a second attack, the other recovered.]

II.—*On the Atheromatous Expression.* By George D. Gibb, M.D. (London Lancet, May 12, 1860, p. 463.)

We have here a record of the observations of one who acknowledges that his attention for a number of years has been directed to the inquiry whether those with whom he was brought in contact in the daily routine of practice, or in the course of his daily perambulations, were characterized by an "atheromatous expression." There is a large number of persons who enjoy, apparently, a fair share of health, and whose appearance to many is that of extremely good health, but who, to an acute observer, have a peculiar expression of countenance, induced, as Dr. Gibb thinks, by changes in the system at large from the influence of fat and its numerous compounds, the invariable result of saccharine conversion in the economy. The pathological phenomena associated with such an alteration of the countenance are mainly due to changes in the blood, leading to the deposition of atheromatous matter in the coats of the blood-vessels. Life becomes jeopardized by the risk of cerebral hemorrhage or intense congestion, by fatal cardiac syncope, or by aneurismal disease in distant parts. The condition of the vessels is never the result of inflammatory action, but is unquestionably due to simple metamorphosis, through the agency of the blood. The characteristic features of the "atheromatous expression," described by Dr. Gibb, are a peculiar greasy appearance of the face, especially about the prominent part of the cheeks and end of the nose; the lips are full, the ala of each nostril is smooth and rounded, and the subcutaneous areolar tissue, being abundantly supplied with adipose matter, gives plumpness and development to the countenance. The skin may be reddish or pinkish, with the small vessels on its surface injected here and there with blood of a bright-red color, sometimes having an irregularly streaked appearance. The eyes have a fatty lustre, and sometimes a simple arcus adiposus or arcus senilis exists, or occasionally a complete annulus of the same kind.

The expression is by no means one of discontent or ill health, and may be seen in those in the prime of life as well as in those of advanced age. "As a rule," says the writer, "which I have worked out by watching a large number of persons thus featured, life is extremely uncertain, and sooner or later becomes the forfeit, to the astonishment of their friends, who had hitherto looked upon them as models of health from their complexion and general appearance. Persons thus featured, may, however, with quiet, care, moderation in living, the avoidance of excitement, etc., go on smoothly and comfortably to a ripe old age." The *calcareo-atheromatous expression*, a modification of the other, is accompanied with an excessive degree of pallor, depending upon the extensive deposition of calcareous or earthy matter in the distal blood-vessels, that is, in the major vessels of the extremities, which become hard cylinders. A distinct bluish-white ring, in these cases, surrounds the entire margins of the cornea. Dr. Gibb describes one case in which the *calcareous expression* alone existed, with an entire absence of atheroma. The heart and all the blood-vessels are not extensively diseased in these affections simultaneously; in the case just cited, for example, the heart not being perhaps involved beyond calcification (not atheromatous degeneration) of the coronary arteries. It is observed that while the body becomes in the condition of advanced senility, the intellectual vigor remains perfect. The subject is one open to further investigations, and worthy of more extended observation.

III.—*Importance of the Functions of the Skin in the Pathology and Treatment of Tubercular Consumption.* By Dr. TOULMIN, of St. Leonard's. (Medical Times and Gazette, April 14, 1860, p. 382.)

The proximate cause of tubercle, is, in all cases, according to Dr. Toulmin, the breathing of impure air, and air in so small a quantity as to render it impure, especially during the night. The rich being subject to phthisis as well as the poor, he drew the attention of the Harveian Society to the importance of the respiratory functions of the skin, as proved by the fact that death resulted from closing the cutaneous pores artificially by varnishing the skin of rabbits and other animals, and suggested that from the coldness of climate and other reasons the better classes of society did not daily wash the whole surface of their bodies, and that the débris of the cuticle and the exuvia momentarily forming on the skin rendered the surface impervious to air. In this mode the same imperfect oxygenation of the blood ensues as is produced in the poorer classes by breathing mephitic air. For the removal of this state of skin, the only means of cure is free diaphoresis by artificial heat, which softens and expels large quantities of sebaceous matter, after the surface has been washed clean with soap and water. Novel doctrines as to the nature and treatment of phthisis were enunciated; the treatment being considered under its hygienic and medical aspects; under the former, the patient was recommended to live in a high, dry, and marine atmosphere on the downs rather than under them; and to be in the open air, devoting himself to suitable athletic exercises. Medically, the treatment was mainly the use of the hot-air bath, the daily anointing of the whole surface with oleaginous matter, the keeping up open counter-irritation by setons or issues, and the use of tonics—each means of medication admirable in itself, but inefficacious if the hot-air bath, followed by aspersion of cold or tepid water, should be omitted.

IV.—*On Contraction and Obliteration of the Aorta near the Junction of the Ductus Arteriosus.* By THOMAS B. PEACOCK, M.D., F.R.C.P., etc. (Brit. and For. Med.-Chir. Review, April, 1860, p. 467.)

All the important facts connected with the pathology of the cases embraced in Dr. Peacock's comprehensive paper are considered in Dr. Packard's analysis, under the department of "Pathological Anatomy." Other facts of historical interest attach to each case, which may be more appropriately arranged in the department of "Practice of Medicine." We have carefully collected these in a table, giving the "local habitation" of each case, and the sources of our information. We thus supply what we believe to be a useful supplementary index of reference to the contents of Dr. Peacock's paper.

### HISTORICAL SKETCH OF ALL THE RECORDED CASES OF OBSTRUCTION

| | Date. | Sex. | Age. | Where occurred. | Described by whom. |
|---|---|---|---|---|---|
| 1 | 1791 | Female | 50 years | Paris | M. Desault |
| 2 | 1814 | Male | 14 " | Glasgow | Dr. Graham |
| 3 | 1818 | Male | 57 " | London (probably) | Sir Astley Cooper and Mr. Travers |
| 4 | 1820 | Female | 17 " | Berlin (probably) | Dr. A. W. Otto |
| 5 | 1827 | Male | 35 " | Germany | M. Albert Meckel |
| 6 | 1828 | Male | 92 " | France | M. Reynaud |
| 7 | 1828 | Male | 40 " | Caen | M. Pellétier |
| 8 | 1831 | Male | 21 " | Manchester | Mr. Jordan |
| 9 | 1834 | Male | 27 " | Dublin | Mr. Nixon |
| 10 | 1834 | Male | 48 " | Paris | M. Le Grand |
| 11 | 1839 | Male | 38 " | Paris | M. Mercier |
| 12 | 1839 | Male | 50 " | Austria | M. Roemer |
| 13 | 1835–42 | Unknown | Unknown | Unknown | M. Cruveilhier |
| 14 | 1840 | Male | Unknown | Ireland | Dr. Houston |
| 15 | 1841 | Female | 7 years | Edinburgh | Dr. Craigie |
| 16 | 1842 | Female | 19 " | Birmingham | Dr. Fletcher |
| 17 | 1842 | Male | Middle aged | Calcutta | Dr. Wise |
| 18 | 1842 | Male | 69 years | Germany | M. Tiedemann |
| 19 | 1844 | Male | 42 " | Prague | M. Hamernjk |
| 20 | 1845 | Male | Unknown | England | Dr. Chevers |
| 21 | 1845 | Male | 22 days | Prague | Dr. Bochdalek |
| 22 | 1845 | Female | 4 years | Prague | Dr. Bochdalek |
| 23 | 1847 | Male | 25 " | London | Mr. Muriel |
| 24 | 1848 | Female | 20 " | London | Dr. Blakiston |
| 25 | 1848 | Male | 48 " | London | Dr. Blakiston |
| 26 | 1848 | Male | 25 " | Prague | M. Hamernjk |
| 27 | 1849 | Male | 14 " | Utrecht | Dr. Van Leeuwen |
| 28 | 1852 | Male | 22 " | Paris | M. Lébert |
| 29 | 1852 | Male | 32 " | Paris | M. Lébert |
| 30 | 1852 | Male | 27 " | Unknown | M. Rokitansky |
| 31 | 1852 | Male | 29 " | Vienna | M. Löbl |
| 32 | 1852 | Male | 57 " | Unknown | M. Rokitansky |
| 33 | 1853 | Male | 45 " | London | Mr. Sydney Jones |
| 34 | 1853 | Female | 56 " | Stuttgart | Dr. A. Härlin |
| 35 | 1856 | Female | 39 " | Paris | M. Dumonpallier |
| 36 | 1857 | Female | 37 " | Rouen | M. Leudet |
| 37 | 1858 | Male | 22 " | London | Dr. Wilks |
| 38 | 1858 | Male | Unknown | London | Mr. John Wood |
| 39 | 1860 | Female | 28 years | Sweden | Hr. Kjellberg |
| 40 | 1860 | Male | 24 " | London | Not yet described |

OF THE AORTA NEAR THE JUNCTION OF THE DUCTUS ARTERIOSUS.

| Authorities. | Remarks. |
|---|---|
| Journal de Chirurgie, tome ii. p. 107, Paris, 1791. | Discovered by M. Paris in a body injected for dissection. |
| Medico-Chirurgical Transactions, vol. v. p. 287, 1814. | Described also by Mr. H. Rainey in the Journal of Corvisart. |
| Surgical Essays, by A. Cooper and B. Travers, part i. p. 103; 1818. | Occurred in the practice of Mr. Winston. |
| Neue seltene Beobachtungen, p. 66; Berlin, 1824. | |
| Meckel's Archiv für Anatomie und Physiologie, p. 345, 1827; Tiedemann, von der Verengung, etc. der Pulsadern, p. 15. | Post-mortem examination performed by M. Herman. |
| Journal Hebd. de Méd., tome i. | Cases 1, 2, and 3 are also referred to by M. Reynaud. |
| Archives Générales de Méd., tome xviii. (16me année,) p. 205, 1828. | Occurred in the practice of M. Trouvé. |
| North of England Medical and Surgical Journal, from Aug., 1830, to May, 1831, vol. i. p. 101. | |
| Dublin Jour. of Med. Science, vol. v. p. 386, 1834. | Cases 1, 2, 3, 6, and 8 are also referred to by Mr. Nixon. |
| Du Rétrécissement de l'Aorte, etc., Paris, 1834; noticed in Gaz. Méd. de Paris, 1834, and Arch. Gén. de Méd., deuxième série, tome viii. p. 528, 1835. | Patient first seen by M. Le Grand and M. Kapeler. Post-mortem examination by M. Amussat. Five of the preceding cases also referred to by M. Le Grand. |
| Bulletin de la Soc. Anat., 14me année, p. 158, 1839. | |
| Med. Jahrb. des Oesterreich. St., 1839; and Tiedemann, op. cit., obs. 1. | Case previously under the care of M. Eichler. |
| Pathological Anat., liv. xl. pl. 3, fig. 3. | |
| Catalogue of the Pathological Museum of the Royal College of Surgeons of Ireland, B. c. 183. | Case previously under care of Dr. Hargraves. |
| Edinburgh Med. and Surgical Jour., vol. lvi. p. 427, 1841. | Cases 1, 2, 3, 4, 5, 6, 8, 9, and 10 are also quoted in this Memoir. |
| Medico-Chirurgical Transactions, vol. xxv. p. 282, 1842. | |
| Transactions of the Medical and Physical Society of Calcutta, vol. viii. p. 384, 1842. | |
| Opus citatum, Beobachtungen, ix. p. 15. | Cases 1 to 17 inclusive are also referred to by M. Tiedemann. |
| Prager Vierteljahrschrift, p. 41, 1844. | |
| London Medical Gazette, vol. xxxvi. (new series, vol. i.) p. 187, 1845. | The preparation is in Guy's Hospital Museum. |
| Vierteljahrschrift, p. 160, 1845. | |
| " " " | |
| Guy's Hospital Reports for 1847, (p. 453.) | |
| Practical Observations on Certain Diseases of the Chest, p. 98, 1848; case xxix. p. 134. | |
| Ibid., case xxx. p. 135. | |
| Vierteljahrschrift, p. 40, 1848. | Occurred in the clinique of Prof. Oppolzer, of Prague, and was correctly diagnosticated by him during life. |
| Nederlandsch Lancet, 2d series, 5th year, No. 2. | Diagnosticated by Dr. Van Leeuwan during life. Cases 11, 19, and 26 are also referred to. |
| Archiv, etc., von Virchow und Reinhardt, s. 327, Berlin, 1852. | Occurred in the practice of M. Louis. |
| Do. do. do. do. do. | Occurred in the practice of M. Barth. |
| Pathological Anatomy, Lond. Edit., 1852. | Occurred in the practice of M. Dlauhy. |
| Rokitansky, Ueber einige der wichtigsten Krankheiten der Arterien, Obs. 20, tab. xiv. B. | Occurred in Prof. Skoda's wards, in Vienna Hospital, and is detailed in Rokitansky's monograph "On Some of the More Important Diseases of the Arteries." |
| Ibid., Obs. 21, tab. xv. | Described in Rokitansky's monograph. |
| Path. Trans., vol. viii., 1856–7, p. 159. | The preparation is retained in the Museum of St. Thomas's Hospital. |
| Archiv, etc., von Virchow, p. 273, Berlin, 1853. | |
| Comptes Rendus des Séances et Mémoires, tome iii. deuxième série, année 1856, p. 273, Paris, 1857; and Gaz. Méd. de Paris, 27ème année, 3ème serie, tome xii. pp. 52, 188, 295. | Occurred in the practice of M. Voillemier, at Hôpital Lariboîssière. Many of the preceding cases are also analyzed in this as well as the next report. |
| Ibid., tome iv. 2ème série, année 1857, p. 63, Paris, 1858; Gaz. Méd. de Paris, 28ème, année 1858, p. 44. | Occurred in M. Leudet's practice at the Hôtel-Dieu of Rouen. |
| Trans. of Path. Soc. of London, vol. x., 1858–9, p. 77. | A patient of Dr. Rees in Guy's Hospital. |
| " " " " " p. 97. | |
| Hygeia, Medicinsk och Farmaceutisk Mundschrift, vol. xxi. No. 6, p. 376. | |
| Brit. and For. Med.-Chir. Rev., April, 1860, p. 480. | A patient of Dr. Barker at St. Thomas's Hosp. |

V.—*Unsuccessful Attempts to produce Variola in the Cow by inoculating with the Virus of True Variola; Perfect Success on using the ordinary Vaccine Virus.* By EPHRAIM CUTTER, M.D., Woburn, Mass. (Boston Med. and Surg. Journal, March 15, 1860.)

A series of experiments upon fifty cows was instituted by Dr. Cutter to test whether, as cow-pox is commonly thought to be small-pox modified and mitigated by a transmission through the system of the cow, it is possible to procure pure primary vaccine virus by simply introducing into the system of the cow the virus of variola from the human subject. Three modes of introducing the variolous matter into cows were used—(*inoculation*):—

1. By quill and puncture with lancets.

2. By rubbing the charged points of quills upon crucial abrasions of the hairless cutis.

3. By introducing, in the form of setons, threads charged with the variolous virus; this being the easiest method.

The experiments were wholly unsuccessful. *Vaccination* on the cow was practiced in the following modes:—

1. By the seton; this was tried twice, but unsuccessfully.

2. By quills; these, if fresh, generally succeeded.

3. By pricking into crucial abrasions of the cuticle, with a lancet, portions of a scab dissolved in water to a pasty consistence. This plan was uniformly successful.

Any one can procure a vaccine pustule on the cow by vaccination, as easily as it can be procured upon the human subject; but Dr. Cutter thinks the virus thus obtained is no better than the ordinary virus in use. Some of the secondary vaccinations with virus from the cow procured pustules normal in appearance, and of a large size, despite a good scar of the primary vaccination; and constitutional symptoms were produced in some cases. Equally good pustules and similar constitutional effects were produced also by the ordinary virus in use. The object of Dr. Cutter's paper is to show that vaccinia is not varioloid, or cow-pox modified small-pox. This, he thinks, is proved by the unsuccessful attempts to produce a normal vaccine pustule by inoculation, while upon the very same animals, by vaccination with the virus in ordinary use, the normal vaccine vesicle has been got easily. Von Bibra states distinctly that the cow-pox and the small-pox are two different diseases; and the asserted fact that persons who have had the small-pox have been successfully vaccinated, seems to substantiate the position assumed by Dr. Cutter.

VI.—*On a form of Hypochondriacal Delirium occurring consecutive to Dyspepsia, and characterized by Refusal of Food.* By DR. L. V. MARCÉ. (Annales Médico-Psychologiques, January, 1860, in the Journal of Psychol. Med., April, 1860.)

Peculiar mental conditions sometimes accompany dyspepsia, and convert the gastric nervous disorder into what Dr. Marcé calls a cerebro-nervous condition. Loss of appetite or the uneasiness caused by digestion may induce in those who are predisposed to insanity from hereditary antecedents, or those who are very impressible by nervous disturbances, a delirious conviction that they cannot or ought not to eat. Under this condition the stomach contents itself with the feeblest nourishment; in one case the daily amount of solid and liquid food taken did not exceed by actual weight 50 grammes. It is true that all the symptoms preceding death from inanition are very strikingly manifested; but as the debility increases, the intellectual energy seems to centre around the functions of the stomach, and the unhappy sufferer becomes incapable of sustaining the least conversation beyond his delirious ideas, regaining a certain amount of energy in order to resist all attempts at alimentation. The physician is at first deceived as to the nature of the affection, and attempts to stimulate the activity of the digestive functions. But when the disease is advanced, it is no longer the stomach that demands attention, for that organ may have perfect digestive powers, which cannot, however, be exercised in the absence of food; it is the delirious idea which constitutes the essence of the malady. The patients are no longer dyspeptics; they are insane. Removal from their habitual domestic circle; intimidation, and even force; the use of the œsophagus sound, and the gradual increase by weight of the daily allowance of food, are the means of treatment suggested by Dr. Marcé, assisted by the employment of bitters, tonics, and a watchful attention to any tendency to relapse.

